# Large scale simulation of pressure induced phase-field fracture propagation using Utopia

**DOI:** 10.1007/s42514-021-00069-6

**Published:** 2021-06-29

**Authors:** Patrick Zulian, Alena Kopaničáková, Maria Giuseppina Chiara Nestola, Andreas Fink, Nur Aiman Fadel, Joost VandeVondele, Rolf Krause

**Affiliations:** 1 Euler Institute, Università della Svizzera italiana, Via la Santa 1, 6962 Lugano-Viganello, Switzerland; 2 Institute of Geochemistry and Petrology, ETH Zurich, Clausiusstrasse 25, 8092 Zurich, Switzerland; 3 Swiss National Supercomputing Centre (CSCS), ETH Zurich, Zurich, Switzerland

**Keywords:** Parallel implementation, Scientific code, Non-convex minimization, Multilevel methods, Phase-field fracture propagation, monolithic solution scheme

## Abstract

Non-linear phase field models are increasingly used for the simulation of fracture propagation problems. The numerical simulation of fracture networks of realistic size requires the efficient parallel solution of large coupled non-linear systems. Although in principle efficient iterative multi-level methods for these types of problems are available, they are not widely used in practice due to the complexity of their parallel implementation. Here, we present Utopia, which is an open-source C++ library for parallel non-linear multilevel solution strategies. Utopia provides the advantages of high-level programming interfaces while at the same time a framework to access low-level data-structures without breaking code encapsulation. Complex numerical procedures can be expressed with few lines of code, and evaluated by different implementations, libraries, or computing hardware. In this paper, we investigate the parallel performance of our implementation of the recursive multilevel trust-region (RMTR) method based on the Utopia library. RMTR is a globally convergent multilevel solution strategy designed to solve non-convex constrained minimization problems. In particular, we solve pressure-induced phase-field fracture propagation in large and complex fracture networks. Solving such problems is deemed challenging even for a few fractures, however, here we are considering networks of realistic size with up to 1000 fractures.

## Introduction

Fractures and fracture networks strongly affect the hydraulic and mechanical response of the underground. This is of particular relevance for geothermal technologies, which aim at producing electricity from deep geothermal resources by enhancing the permeability of a geothermal reservoir to obtain a sufficiently large heat flux on interior surfaces (Chen et al. 2018; Samin et al. 2019). In numerical simulations, realistic fracture networks are usually challenging to represent with a discrete geometry (i.e., a mesh), or even impossible at the macro-scale. Phase-field approaches for fracture allow for modelling and simulating the fracture initiation, propagation, and interaction without the need of explicitly representing the fracture surface (Miehe et al. 2010b).

The basic idea of this method is to model systems with sharp interfaces or fractures using a continuous variable, called the phase-field, that allows for incorporating the presence of fractures into a given system through a *smooth* transition between two states, i.e. damaged and not damaged. The first numerical implementation of a variational phase-field approach was presented in Bourdin (2007). Miehe et al. (2010b, 2010a) enhanced the underlying mathematical model and introduced thermodynamically consistent, rate-independent formulation. Since then, the phase-field approach has become popular and it has been extended in many directions, including dynamic models (Bourdin et al. 2011), generalization to large deformations (Hesch and Weinberg 2014; Bilgen et al. 2019), adaptive fourth-order models (Goswami et al. 2020), or anisotropic models for a fracture of fiber-reinforced matrix composites (Denli et al. 2020). For further details, we refer the interested reader to the review provided in De Lorenzis et al. (2020).

Despite the popularity of the phase-field fracture models in recent years, their applicability is fairly limited to the small scale problems due the following limitations.

1. First, high-resolution meshes are required to resolve the localized damage, which leads to simulations with a huge number of degrees of freedom.
2. Secondly, solving the resulting problems numerically is challenging as the underlying energy functional is non-convex and therefore the standard solution strategies, such as Newton’s method, typically fail to converge.

As a consequence, the design of the large-scale fracture simulation framework requires both, highly-scalable finite element implementation of the fracture model and the globally convergent, yet scalable, solution strategy.

The majority of the model implementations in the literature relies on in-house finite element codes, based for example on the environment Matlab (Nguyen et al. 2015, 2017; Hesch et al. 2017). First commercial implementations appeared in software such as Abaqus (Liu et al. 2016; Molnár and Gravouil 2017; Msekh et al. 2015) and COMSOL (Zhou et al. 2018). More recently, several open source codes were reported, for example Farrell and Maurini (2017) and Li et al. (2016) use the finite element framework FEniCS (Logg 2007) to implement a quasi-static and dynamic model for brittle fractures, respectively. The implementation documented in Heister et al. (2015), and Klinsmann et al. (2015) relies on the package Deal II. Bangerth et al. (2007) and supports adaptive mesh refinement strategies. The MOOSE environment Gaston et al. (2009) served as a base for the implementation reported in Chakraborty et al. (2016a, 2016b). The results obtained in Kuhn et al. (2015), Steinke et al. (2016) were produced using FEAP (Taylor 2020). Additionally, the JIVE framework (Group research 2015) was utilized in May et al. (2015), while the package NUTIL (van Zwieten 2018) was used in Singh et al. (2016). A GPU implementation was presented in Ziaei-Rad and Shen (2016), where the authors demonstrate a speedup factor of 12 for simulations with around 2.5 million degrees of freedom (dofs). A thread scalable implementation based on the Kokkos library (Edwards et al. 2014) was presented in Tupek (2016) for cohesive fracture.

Several aforementioned codes are implemented on the top of parallel finite element framework. However, their applicability to solve large-scale problems is often limited by the convergence and the scaling properties of a utilized solution strategy. The widely adopted solution strategy in the literature is the alternate minimization (Bourdin et al. 2011; Farrell and Maurini 2017). The main idea behind this method is to minimize the energy functional successively for the displacements and phase-field variables. This gives rise to two convex minimization sub-problems, which are then alternatively solved until convergence is reached. Although solving the convex sub-problems is fairly straightforward, the overall convergence speed of the method can be erratic (Farrell and Maurini 2017). Moreover, the scalability properties of this approach are also limited, as the number of variables, and consequently, the size, of the two sub-problems differs. In this regard, the monolithic approach, where both sub-problems are solved simultaneously, can be computationally more efficient. Several attempts have been made to enhance the convergence and the robustness of the method, for instance path-following strategies (Singh et al. 2016), line-search methods (Gerasimov and Lorenzis 2016), primal-dual algorithms (Heister et al. 2015), modified Newton’s method (Wick 2017), quasi-Newton’s method (Wu et al. 2020), or fast Fourier transform (FFT) (Chen et al. 2019).

The use of these methods to solve large scale problems is mainly limited by the use of direct linear solvers for the solution of the arising linear systems. To this aim, multilevel strategies have been employed as an inner linear solver, due to their optimal complexity. In particular, a geometric multigrid method was applied in Bilgen et al. (2018) showing scalability up to 300 processes, while matrix-free multigrid was used in Jodlbauer et al. (2019), demonstrating scalability up to 128 cores. Alternative approaches, based on truncated non-smooth non-linear monotone multigrid, were used in Kienle et al. (2018), where authors obtained a significant improvement in terms of computational time, but the parallel performance was not reported. More recently, nonlinear multilevel method based on the trust-region method, called Recursive Multilevel trust-region (RMTR) Gratton et al. (2008a); Groß and Krause (2009), has been developed in Kopaničáková and Krause (2020). RMTR for phase field ensures global convergence and has been shown to scale up to 300 processes.

In this work, we provide large scale implementation of the phase-field fracture approach. Our simulation framework is designed to solve large scale problems inspired by real-world applications, e.g., pressure induced fracture propagation in geothermal reservoir (Wick et al. 2016; Yoshioka and Bourdin 2016; Mollaali et al. 2019). In particular, motivated by the promising properties of the RMTR method, shown in Kopaničáková and Krause (2020), we extended the approach to complex scenarios with hundreds of fractures in three-dimensions and with thousands of fractures in two-dimensions. To our knowledge, this is the first time that the phase-field approach is employed for such complex, large scale scenarios.

The biggest challenge in designing the long lasting simulation code is to keep up with constantly changing technology, which gives rise to new programming paradigms and new languages. For example, the advent of GPGPU induced languages such as Cuda (Nickolls et al. 2008) and OpenCL (Khronos OpenCL Working Group 2008), which led to the creation of new software libraries such as CuBLAS (Nvidia 2008) and ViennaCL (Rupp et al. 2016). With such new developments, scientific-computing software libraries need to be constantly updated or rewritten. In order to avoid changes in high-level algorithms, such as non-linear solution strategies, or finite element analysis, several application codes are developed on top of a portable interface that fits many current and possibly future requirements [e.g., PETSc (Balay et al. 1997, 2019), Trilinos (Heroux et al. 2003), and Kokkos (Edwards et al. 2014)]. Software libraries such as Deal.II (Bangerth et al. 2007), LibMesh (Kirk et al. 2006), Dune (Blatt et al. 2016), and MOOSE (Gaston et al. 2009) rely on high level abstractions on top of existing linear algebra and non-linear solution strategies codes, and allow choosing, to some degree, the underlying implementation.

However, a clear separation of frontend programming and the backend implementation would help in keeping up with even new technologies or upcoming and yet unknown paradigm shifts. A best-case scenario allows us to never touch the frontend code and implement new backends based on these new technological advancements.

To this end, a possible solution is to exploit scripting facilities for completely decoupling the application behavior from its actual implementation. This solution has the advantage of hiding the complexity of parallel software to which the average, casual, or opportunistic (Brandt et al. 2008) user is not supposed to be exposed. The idea is that the scripting code is translated to behavior which is implemented in another lower-level language. This enables users to write a few lines of very powerful code without the overhead of learning how to use new complex parallel scientific codes. A very specific form of scripting language is usually referred to as domain specific language (DSL). This specificity, while reaching the aforementioned objectives, has a twofold advantage. First, it enables a simple description of a specific problem since most implementation details can be hidden. Second, it allows exploiting complex functionalities and performance critical optimizations. Notable examples related to finite element software, are FEniCS’ unified form language (Logg 2007; Rathgeber et al. 2016), FreeFEM (Hecht 2012), and Liszt (DeVito et al. 2011).

In DSLs lower-level abstractions are purposefully inaccessible because the actual algorithms are implemented in a different language, such as C++. This is a problem when a DSL misses a functionality, since adding it would require accessing the underlying back-end which may be either closed source or very complex. In contrast, embedded domain-specific languages (eDSL) [e.g., CULA (Humphrey et al. 2010), Feel++ (Prud’homme et al. 2020), OpenFOAM (Weller et al. 1998), Sundance (Long et al. 2010)] use the same language and compiler for both the “scripting” layer and the implementation of the back-end. For this reason, eDSLs have the opportunity to provide the right balance between abstraction and direct access to the back-end data-types and algorithms.

In this work, we introduce the open-source C++ library Utopia (Zulian et al. 2016), which currently provides a eDSL-like uniform interface to the PETSc algebra, and Tpetra from the Trilinos library. The main goal of Utopia is to achieve a set of high-level interfaces with will allow to never fully commit to particular software/hardware and adapt to the ever-evolving HPC technologies. Unfortunately, it is the case that specificity is required for achieving performance in certain applications. Hence, while the largest part of the code is designed to be generic, certain routines are implemented ad-hoc. Here, we present our implementation of the phase-field fracture simulation framework, its portable components as well as the ad-hoc ones targeting CPU architectures.

The five main contributions of this article are:

1. the first introduction of the open-source C++ library Utopia (Zulian et al. 2016);
2. efficient open-source finite element code for phase-field fracture simulations;
3. the only parallel open-source code of the RMTR method, an efficient globally convergent nonlinear multilevel solution strategy for non-convex constrained minimization problems;
4. large scale simulations of pressure-induced fracture propagation of stochastic fracture networks, considering realistic and complex scenarios up to 1000 fractures;
5. strong and weak scaling studies up to 9216 MPI processes and $1.9 \times 10^8$ degrees-of-freedom of the proposed algorithmic framework and its CPU-tailored implementation using Utopia.

We start by describing the pressure-induced phase-field fracture model (Sect. 2), and the recursive multilevel trust-region strategy (Sect. 3), adopted to solve the arising nonlinear systems. Next, we provide an overview of our software library and a detailed description of the developed code (Sect. 4). Then, we validate the implementation of the phase-field fracture model and present numerical experiments with complex fracture networks for applications in geoscience (Sect. 5). Furthermore, we demonstrate the strong and weak scaling performance properties of our code using the Piz Daint super-computing machine (Sect. 6). Finally, we provide concluding remarks and describe future work (Sect. 7).

## Pressure induced phase-field fracture model

In this section, we briefly review pressure-induced fracture processes modeled using the second-order phase-field formulation for brittle fracture. Our presentation focuses on the quasi-static time-discrete setting. A pseudo-time step $t=1, \ldots , T$, is used to index the deformation state in the loading process. We denote the computational domain by $\varOmega \in {\mathbb {R}}^d, d=2,3$, representing a *d*-dimensional solid with internal fracture $C \subset {\mathbb {R}}^{d-1}$, which evolves during the loading process. The boundary $\partial \varOmega$ of the domain $\varOmega$ is decomposed into two non-overlapping parts, $\varGamma _D$, $\varGamma _N$, where Dirichlet and Neumann boundary conditions are prescribed, respectively. Additionally, we set $\partial \varOmega _N = \varGamma _N \cup \partial C$.

In this work, we assume that the body $\varOmega$ shows linear elastic behaviour, with the strain energy density function defined as: $\psi _e( \varvec{\varepsilon }({\mathbf {u}}) ):= 0.5 \, \lambda (\text {tr}(\varvec{\varepsilon }({\mathbf {u}})))^2 + \mu \varvec{\varepsilon }({\mathbf {u}}):\varvec{\varepsilon }({\mathbf {u}})$, where $\mu , \lambda$ are the Lamè parameters, ${\mathbf {u}}: \varOmega \rightarrow {\mathbb {R}}^d$ represents the displacement vector field and $\varvec{\varepsilon }({\mathbf {u}}) := \text {sym}(\nabla {\mathbf {u}})$ is the strain tensor. Furthermore, we prescribe a given pressure $p: \varOmega \rightarrow {\mathbb {R}}$, over the domain $\varOmega$ to only induce fracture propagation. We remark, that this work focuses only on fracture propagation, i.e. we assume that pressure *p* is given a priori. The reliability of the phase-field fracture model could be improved by incorporating the poroelasticity equations such as Biot’s equations (Mikelić et al. 2015a). This would allow for simulating induced hydraulic fracturing in a poroelastic medium rather than in an elastic medium.

### Variational approach to fracture

The variational approach proposed by Francfort and Marigo (1998) formulates brittle fracture as a minimization problem for an energy functional consisting of the elastic energy of the cracked solid, the energy dissipated in the fracture, and the traction forces; thus

1 $$\begin{aligned} \begin{aligned} E({\mathbf {u}}, C, p)&:= \int _{\varOmega {\setminus } C} \psi _e( \varvec{\varepsilon }({\mathbf {u}}) ) \ d\varOmega \\&\quad + \mathcal {G}_c S^{d-1}(C) - \int _{\partial _N \varOmega } {\bar{\mathbf {t}}} \cdot {\mathbf {u}}\ ds, \end{aligned} \end{aligned}$$

where $\mathcal {G}_c > 0$ denotes fracture toughness and ${\bar{\mathbf {t}}}$ stands for the traction forces. The symbol $S^{d-1}(C)$ in (1) denotes the Hausdorff surface measure of fracture set *C*, i.e. $S^{d-1}(C)$ represent length or the surface area of fracture *C*, when $d=2,3$, respectively. Note, that the traction forces ${\bar{\mathbf {t}}}$ constitute of two parts

2 $$\begin{aligned} \int _{\partial _N \varOmega } {\bar{\mathbf {t}}} \cdot {\mathbf {u}}\ ds = \int _{\varGamma _N} {\bar{\mathbf {t}}}_{\varOmega } \cdot {\mathbf {u}}\ ds - \int _{\partial C} p \ \mathbf {n}\cdot {\mathbf {u}}\ ds, \end{aligned}$$

where ${\bar{\mathbf {t}}}_{\varOmega }$ is traction force applied at the domain boundary $\varGamma _N$ and $\mathbf {n}$ is unit vector normal to the fracture surface. The last term in (2) represents a force introduced by the pressure *p* inside of the fracture, which is applied on a surface.

The direct minimization of the energy functional (1) is computationally prohibitive, as the fracture surface, *C* is not known a priori. To overcome this difficulty, Bourdin (2007) propose to utilize a regularization strategy initially developed by Ambrosio and Tortorelli (1992) for image-segmentation. The regularization strategy introduces a smooth scalar field, called phase-field $c: \varOmega \rightarrow [0,1]$, which characterizes the material state of the domain $\varOmega$. In particular, the value $c=0$ indicates an intact solid, $c=1$ denotes the fractured or broken state, while $c \in (0,1)$ constitute smooth transition zones between the two limit states. Using the phase-field *c*, we can replace the fracture energy in (1) by its volumetric approximation, i.e.,

3 $$\begin{aligned} \mathcal {G}_c S^{d-1}(\varGamma ) \approx \frac{\mathcal {G}_c}{c_w} \bigg ( \frac{w(c)}{l_s} + l_s \left| \nabla c \right| ^2 \bigg ) d\varOmega , \end{aligned}$$

where the length-scale parameter $l_s$ controls the thickness of the transition zone between the material states. The function *w* defines a decaying profile of the phase-field *c*, while $c_w:=4 \int _{0}^1 \sqrt{w(c)} \ dc$ is an induced normalization constant. Taking into account (3), we can reformulate (1) as

4 $$\begin{aligned} E({\mathbf {u}}, c, p)&:= \int _{\varOmega } g(c) \ \psi _e( \varvec{\varepsilon }({\mathbf {u}})) + \frac{\mathcal {G}_c}{c_w} \left( \frac{w(c)}{l_s} + l_s \left| \nabla c \right| ^2 \right) d\varOmega \nonumber \\&\quad - \int _{\partial _N \varOmega } {\bar{\mathbf {t}}} \cdot {\mathbf {u}}\ ds, \end{aligned}$$

where *g* is a degradation function, which accounts for the loss of stiffness in the fracture.

Several choices of *g*, *w* and $c_w$ are used in the literature, leading to various phase-field fracture formulations (Kuhn et al. 2015; Sargado et al. 2018). In this work, we follow Bourdin et al. (2000), Miehe et al. (2010b) and employ $g(c):=(1-c)^2$, $w(c)=c^2$ and $c_w=2$, resulting in the widely used *AT-2* phase-field fracture model proposed in Ambrosio and Tortorelli (1990). Given these particular choices, it is possible to asymptotically show via $\varGamma$-convergence, that the minimizer of (4) tends to a minimizer of (1), as $l_s \rightarrow 0$, see Giacomini (2005).

In the next step, we reformulate the fracture surface integral from (2), into a computationally acceptable form, which does not include $\partial C$. We follow Mikelic et al. (2014), Mikelić et al. (2015a, 2015b) and employ Gauss’ divergence theorem for extending the pressure *p* to the entire domain, thus

$$\begin{aligned} \int _{\partial C} p \ \mathbf {n}\cdot {\mathbf {u}}\ ds = \int _{\varOmega } g(c) \nabla \cdot (p \ {\mathbf {u}}) \ d \varOmega - \int _{\partial \varGamma _N} p \ \mathbf {n}\cdot {\mathbf {u}}\ ds. \end{aligned}$$

Here, the degradation function *g*(*c*) ensures that the integration is performed only over the intact part of the domain $\varOmega$. Finally, the energy functional (1) can be recast into the following form:

5 $$\begin{aligned} \begin{aligned} E({\mathbf {u}}, c, p)&:=\int _{\varOmega } g(c) \ \psi _e( \varvec{\varepsilon }({\mathbf {u}})) + \frac{\mathcal {G}_c}{c_w} \bigg ( \frac{w(c)}{l_s} + l_s \left| \nabla c \right| ^2 \bigg ) d\varOmega \\& \quad - \int _{\varGamma _N} {\bar{\mathbf {t}}}_{\varOmega } \cdot {\mathbf {u}}\ ds - \int _{\varOmega } g(c) \nabla \cdot (p {\mathbf {u}}) \ d \varOmega \\&\quad + \int _{\partial \varGamma _N} p \mathbf {n}\cdot {\mathbf {u}}\ ds, \end{aligned} \end{aligned}$$

which can be employed in practical algorithms.

### Minimization problem

The state of the system, defined by the displacement ${\mathbf {u}}$ and the phase-field *c*, is characterized at each loading step as minimizer of the following minimization problem: find $({\mathbf {u}}, c) \in \mathbf {V}_u^t \times V_c$, such that

6 $$\begin{aligned} ({\mathbf {u}}, c) \in \text {arg \ min} \ E({\mathbf {u}}, c, p), \end{aligned}$$

where the energy functional $E({\mathbf {u}}, c, p)$ is as defined in (5). The admissible space for the displacement field is defined as $\mathbf {V}_u^t := \{ {\mathbf {u}}\in \mathbf {H}^1(\varOmega ) \ | \ {\mathbf {u}}= \mathbf {g}^t \ \text {on} \ \varGamma _D \}$. Here, $\mathbf {H}^1(\varOmega ):= [H^1(\varOmega )]^d$, with $H^1$ denoting the standard Sobolev space of weakly differentiable functions in $L^2$ with one weak derivative also in $L^2$. We remark that the definition of the space $\mathbf {V}_u^t$ incorporates the time-dependent Dirichlet boundary condition ${\mathbf {g}}^t$. The admissible space for the phase-field is defined as the following convex cone:

7 $$\begin{aligned} V_c := \left\{ c \in H^1(\varOmega ) : c^{t-1} \le c \le 1 \ \text {a.e.\; \ in} \ \varOmega \right\} , \end{aligned}$$

where $c^{t-1}$ represents phase-field obtained in the previous time-step. The box constraint $c^{t-1} \le c \le 1$ from (7) ensures the irreversibility condition and prevents the crack from self-healing.

We discretize our problem using the first-order Lagrangian finite elements. In the remainder of this work, we focus on the numerical solution of (6). This task is numerically challenging and computationally demanding as we have to solve a large-scale, non-convex, constrained, ill-conditioned minimization problem for every loading time-step *t*.

## Multilevel trust-region method

The minimization problem (6) can be expressed in the following abstract form:

8 $$\begin{aligned} \begin{aligned} \min _{x \in {\mathbb {R}}^{n}} f({\mathbf {x}}), \\ \text {such that} \, {\mathbf {x}}\in \mathcal {F}, \end{aligned} \end{aligned}$$

where $f: {\mathbb {R}}^{n} \rightarrow {\mathbb {R}}$ denotes the non-convex coupled energy functional (5) after finite element discretization. The solution vector ${\mathbf {x}}\in {\mathbb {R}}^{n}$ represents the combined displacement and phase-field coefficients. The feasible set $\mathcal {F}:= \{ {\mathbf {x}}\in {\mathbb {R}}^{n} \ | \ {\mathbf {l}}\le {\mathbf {x}}\}$ is defined such that irreversibility condition from (7) is satisfied.

We minimize (8) using the recursive multilevel trust-region method (RMTR) (Gratton et al. 2008a, b; Groß and Krause 2009). In particular, we employ the variant proposed in Kopaničáková and Krause (2020), which was specially designed to solve minimization problems arising from phase-field fracture simulations. By design, the RMTR employs a hierarchy of *L* levels. Each level *l*, where $l=1, \dots , L$, is associated with the minimization of some level-dependent objective function $h^l: {\mathbb {R}}^{n^l} \rightarrow {\mathbb {R}}$, where $n^{l+1} \ge n^{l}$. The transfer of data between subsequent levels of the multilevel hierarchy is achieved using three transfer operators. The prolongation operator ${\mathbf {I}}^l: {\mathbb {R}}^{n^l} \rightarrow {\mathbb {R}}^{n^{l+1}}$ interpolates the corrections from level *l* to level $l+1$. Its adjoint, the restriction operator ${\mathbf {R}}^l := ({\mathbf {I}}^l)^T$, transfers the gradients to the subsequent coarser level. Following Groß and Krause (2009), we additionally employ a projection operator ${\mathbf {P}}^l: {\mathbb {R}}^{n^{l+1}} \rightarrow {\mathbb {R}}^{n^{l}}$ for transferring iterates from level $l+1$ to level *l*.

### RMTR algorithm

The RMTR algorithm is considered in its standard V-cycle form. Through the following paragraphs, we use subscripts and superscripts to specify the iteration number and the given level, respectively. For instance, the symbol ${\mathbf {x}}^l_{i}$ denotes the solution vector on level *l* during iteration *i*.

Each V-cycle consists of a downward and an upward phase. The downward phase starts on the finest level, $l=L$, with an initial iterate ${\mathbf {x}}_0^L$ and passes through all levels of the multilevel hierarchy until the coarsest level, $l=1$, is reached. On each level, the algorithm performs a pre-smoothing step to improve the current iterate ${\mathbf {x}}_{0}^l$. This is done by minimizing the level-dependent minimization problem, see Sect. 3.2. The minimization on a given level is performed only approximately, by employing $\mu _1$ iterations of the trust-region method. The obtained approximate minimizer, ${\mathbf {x}}_{\mu _1}^L$, is then used to initialize the solution vector on the next coarser level, i.e., ${\mathbf {x}}_{0}^{L-1} := {\mathbf {P}}^{L-1} {\mathbf {x}}_{\mu _1}^L$. We repeat this process recursively until the coarsest level is reached.

Once the coarsest level is reached, we again approximately minimize the level-dependent minimization problem to obtain an updated coarse grid iterate ${\mathbf {x}}_{\mu ^1}^1$. After obtaining an updated iterate ${\mathbf {x}}_{\mu ^1}^1$, the RMTR algorithm initiates the upward phase of the V-cycle. An upward phase is associated with the return to the finest level of the multilevel hierarchy while passing through all intermediate levels. Starting on the coarsest level, we compute each coarse grid correction as the difference between the initial and final iterate on the given level, thus as ${\mathbf {x}}_{\mu ^{l-1}}^{l-1} - {\mathbf {x}}_{0}^{l-1}$. This coarse grid correction is then prolongated to the subsequent finer level, e.g. ${\mathbf {s}}_{\mu _1+1}^{l} := {\mathbf {I}}^{l-1}({\mathbf {x}}_{\mu ^{l-1}}^{l-1} - {\mathbf {x}}_{0}^{l-1})$. As common in the trust-region algorithms, the quality of the prolongated coarse grid correction, ${\mathbf {s}}_{\mu _1+1}^{l}$, has to be assessed before it is accepted. To this aim, we define a multilevel trust-region ratio as

9 $$\begin{aligned} \rho ^l := \frac{h^l({\mathbf {x}}_{\mu _1}^l) - h^l({\mathbf {x}}_{\mu _1}^l + {\mathbf {s}}_{\mu _1}^l)}{h^{l-1}({\mathbf {x}}_{0}^{l-1}) - h^{l-1}({\mathbf {x}}_{\mu ^{l-1}}^{l-1})}, \end{aligned}$$

where $\mu ^l$ collectively denotes a sum of all iterations taken on a given level *l*. The positive values of $\rho ^l$ imply a decrease in the fine level objective function $h^l$, therefore it is safe to accept ${\mathbf {s}}_{\mu _1+1}^{l}$. In contrast, small or negative values of $\rho ^l$ suggest that there is no good agreement between fine and coarse level models, therefore ${\mathbf {s}}_{\mu _1+1}^{l}$ has to be rejected.

To this end, the RMTR algorithm performs $\mu _2$ smoothing steps to improve the current solution on a given level *l*. This process is again repeated on every level of the multilevel hierarchy until we reach the finest level. The outlined process is summarized in Algorithm 1.

### Level-dependent minimization problems

On each level *l*, the RMTR method minimizes some level-dependent minimization problem (LDMP). On the finest level, the LDMP is identical with (8), while on all other levels ($l<L$), the LDMP is constructed as follows:

10 $$\begin{aligned} \begin{aligned}&\min _{{\mathbf {s}}^l \in {\mathbb {R}}^{n^l}} h^l({\mathbf {x}}^l + {\mathbf {s}}^{l}) :={\tilde{f}}^l({\mathbf {x}}^l) + \langle \delta {\mathbf {g}}, {\mathbf {s}}^{l} \rangle + 0.5 \langle {\mathbf {s}}^{l}, \delta {\mathbf {H}}{\mathbf {s}}^{l} \rangle , \\&\text {subject to} \ {\mathbf {x}}^l + {\mathbf {s}}^{l} \in \mathcal {F}^l, \end{aligned} \end{aligned}$$

where $h^l$ and $\mathcal {F}^l$ denote the level-dependent objective function and feasible set, respectively. The role of level-dependent feasible set $\mathcal {F}^l$ is two-fold. On the one hand, it ensures that the iterates produced by the RMTR method satisfy the variable bounds. On the other hand, the definition of $\mathcal {F}^l$ also controls the size of all corrections taken on a given level *l*, which is necessary to ensure global convergence (Gratton et al. 2008a). The rigorous details about how to construct $\mathcal {F}^l$ can be found in Gratton et al. (2008a), Kornhuber (1994), and Kopaničáková and Krause (2020).

The definition of the function $h^l$ consists of three terms. The first term, ${\tilde{f}}^l({\mathbf {x}}^l)$, expresses the modified energy functional (4). This modification was suggested in Kopaničáková and Krause (2020) for capturing fine-level fracture on the coarser levels. In this work, we discretize the problem using the finite element method. Therefore, the numerical evaluation of ${\tilde{f}}^l({\mathbf {x}}^l)$ and its derivatives requires the computation of the numerical quadrature.

The terms $\delta {\mathbf {g}}\in {\mathbb {R}}^{n^l}$ and $\delta {\mathbf {H}}\in {\mathbb {R}}^{n^l \times n^l}$ in (10) are defined as following

11 $$\begin{aligned} \delta {\mathbf {g}}:=\; & {} {\mathbf {R}}^l \nabla h^{l+1}({\mathbf {x}}_{\mu _1}^{l+1}) - \nabla {\tilde{f}}^l({\mathbf {x}}^l_0), \nonumber \\ \delta {\mathbf {H}}:=\; & {} {\mathbf {R}}^l \nabla ^2 h^{l+1}({\mathbf {x}}_{\mu _1}^{l+1}) \ {\mathbf {I}}^l - \nabla ^2 {\tilde{f}}^l({\mathbf {x}}^l_0) \end{aligned}$$

and ensure that the first and second order behavior of the $h^l$ and $h^{l+1}$ is similar in the neighborhood of ${\mathbf {x}}_0^l$ and ${\mathbf {x}}_{\mu _1}^{l+1}$. Although the terms $\delta {\mathbf {g}}$ and $\delta {\mathbf {H}}$ are evaluated only once during the V-cycle, their computation is costly. In particular, the evaluation of the $\delta {\mathbf {H}}$ term requires evaluation of the Hessian on level *l* and $l+1$. We can decrease the amount of the Hessian assembly calls by incorporating the lagging strategies into our implementation. In particular, we evaluate the $\delta {\mathbf {H}}$ term from (11) restricting the Hessian evaluated during the pre-smoothing step, i.e., $\delta {\mathbf {H}}:= {\mathbf {R}}^l \nabla ^2 h^{l+1}({\mathbf {x}}_{\mu _1-1}^{l+1}) \ {\mathbf {I}}^l - \nabla ^2 {\tilde{f}}^l({\mathbf {x}}^l_0)$. We note, that this modification slightly worsens the convergence rate of the RMTR method, but offers speed-up in terms of the computational time.

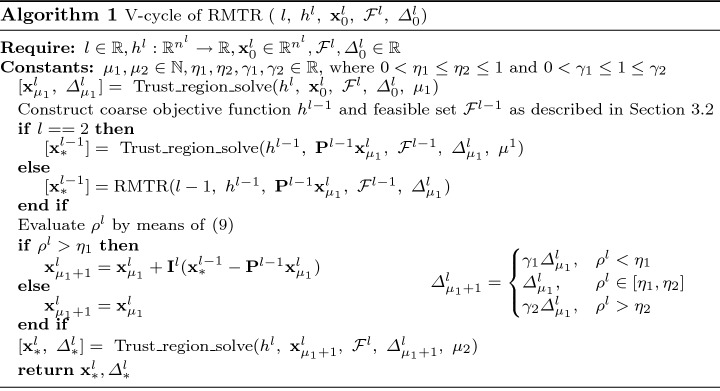



#### Smoothing and coarse grid solve (trust-region method)

We minimize the level-dependent minimization problem (10) using a trust-region method Conn et al. (2000). The following exposition omits using superscript related to a given level *l*, as all quantities are considered to be on the same level. At each iteration *i*, the trust-region method approximates the objective function *h* by quadratic model $m_i$, defined around current iterate ${\mathbf {x}}_i$. The model $m_i$ is considered to be an adequate representation of *h* only in a certain region, called the trust-region $\mathcal {B}_i := \{ {\mathbf {x}}_i + {\mathbf {s}}\in {\mathbb {R}}^n \ | \ \Vert {\mathbf {s}}\Vert _{\infty } \le \varDelta _i \}$, defined by the trust-region radius $\varDelta _i > 0$. The search direction ${\mathbf {s}}_i$ is then determined by solving the trust-region sub-problem:

12 $$\begin{aligned} \underset{{\mathbf {s}}_i \in {\mathbb {R}}^n}{\text {min}} \ m_i({\mathbf {s}}_i )&:= h({\mathbf {x}}_i) + \langle \nabla h({\mathbf {x}}_i), {\mathbf {s}}_i \rangle + \frac{1}{2} \langle {\mathbf {s}}_i, \nabla ^2 h({\mathbf {x}}_i) \ {\mathbf {s}}_i \rangle , \nonumber \\&\quad \text {such that} \ \ {\mathbf {x}}_i + {\mathbf {s}}_i \in \mathcal {F}, \Vert {\mathbf {s}}_i \Vert _{\infty } \le \varDelta _i. \end{aligned}$$

The first constraint in (12) ensures the feasibility of the iterates through the solution process, while the second constraint controls the size of the search direction ${\mathbf {s}}_i$. Before the obtained search direction ${\mathbf {s}}_i$ is used to update the current iterate ${\mathbf {x}}_i$, we need to assess its quality. The convergence control is performed using the trust-region ratio

13 $$\begin{aligned} \rho _i = \frac{h({\mathbf {x}}_i) - h({\mathbf {x}}_i + {\mathbf {s}}_i)}{m_i(\varvec{0}) - m_i({\mathbf {s}}_i) }, \end{aligned}$$

which describes the agreement between the actual reduction in the objective function and the predicted reduction obtained by the quadratic model $m_i$. The value of $\rho _i$ close to unity indicates good agreement between $f_i$ and the model $m_i$. Hence, it is safe to accept ${\mathbf {s}}_i$, i.e. ${\mathbf {x}}_{i+1} = {\mathbf {x}}_i + {\mathbf {s}}_i$, and expand the trust-region radius. In contrast, if the value of $\rho _i$ is negative or close to zero, we must reject ${\mathbf {s}}_i$, i.e. ${\mathbf {x}}_{i+1}={\mathbf {x}}_i$, and shrink the trust-region.

Solution of trust-region subproblem Each iteration of the TR method requires solution of the constrained quadratic minimization (QP) problem (12). The arising QP problems can be solved approximately, as long as the obtained minimizer satisfies the so-called sufficient decrease condition (Conn et al. 2000). Our choice of QP solver varies for different levels of the multilevel hierarchy. In particular, on the coarsest level, we minimize (12) using modified proportioning with reduced gradient projection (MPRGP) method Dostál (2016).

On all the other levels, we employ only few steps of the projected Gauss–Seidel (PGS) method, as it is known to have good smoothing properties (Briggs et al. 2000; Hackbusch 1985). Since the Gauss–Seidel method is naturally a sequential algorithm, we employ its parallel variant, the hybrid Jacobi projected-Gauss–Seidel (HJPGS) method (Adams et al. 2003). More specifically, we use the symmetric version of the HJPGS where both forward and backward substitution are performed. Our implementation of the HJPGS utilizes a copy of diagonal and off-diagonal entries of the local Hessian block into separate arrays. This is mainly done in order to avoid checking if the current row is equal to the current column. We achieve around $2\times$ speed-up compared to the version without a copy. In addition, we perform local iterations of the smoother without synchronization in order to reduce the ratio between computation and communication.

## Parallel implementation with Utopia

### Hardware portability and software maintainability

The first goal of Utopia is the separation of model and computation (similar to DSLs) and its main purpose is advanced parallel algebra (linear and non-linear). By exploiting meta-programming facilities in combination with expression templates (Iglberger et al. 2012; Veldhuizen 1995), Utopia can easily be integrated with any other existing parallel algebra library, hence it is mostly independent from technological changes. The limit case is the avoidance to commit to any specific back-end. This will allow libraries to be unbound by the lifetime and support of the their dependencies although still take advantage of current development efforts.

The second goal is to provide a uniform interface to lower-level technologies [e.g., Kokkos, RAJA (Beckingsale et al. 2019), or SyCL (Bader et al. 2019)]. In fact, the Utopia library is designed and developed for providing a balance between abstraction and low-level access without sacrificing performance. It aims at an organic integration with existing codes without creating barriers between abstractions and implementation. The use of static polymorphism allows the mixture of front-end code with decorated parallel device code without unnecessarily exposing back-end specific primitives or having to write specialized code. High level and lower level abstractions, as well as raw data are accessible to the user at any time. This allows users to extend their code with possibly missing functionalities by manipulating lower-level abstractions and eventually even the low-level data (and back-end) directly. The flexible design of Utopia allows for adding these features in a straightforward way to future releases without changing the high-level interfaces.

The third goal is to reduce the overhead of the front-end and allow to exploit available functionalities of the different back-ends as good as possible. To this end, the use of static polymorphism allows to avoid the performance-overhead associated with virtual tables, and specific evaluation routines can be specialized by exploiting partial/full specialization.

These design goals in combination with the development driven by challenging application codes such as phase-field methods for fractures, allows Utopia to converge towards a hardware portable and maintainable HPC library.

### Algebra

Consistent with the general design goal, the Utopia–algebra library is divided into two main layers the front-end and the back-end. In the front-end every algebraic object, algorithm, or operation is described by C++ classes. Tensor objects, such as matrices and vectors, are tied to a specific back-end at compile time. For instance, PetscMatrix and PetscVector are used when the PETSc backend is chosen for our computations, while TpetraMatrix and TpetraVector are used when the Trilinos backend is chosen instead. Expression types are generated by means of standard operators such as *+*, *-*, *, and */*. In fact, expressions are evaluated in a lazy way only when they are assigned to a concrete tensor type. This allows us to independently specialize the evaluation of composite expressions based on the available back-end algorithms.

More complex algorithms are implemented using classes and they can be realized either using the front-end or the back-end. For instance, the MPRGP and RMTR algorithms are implemented using exclusively the front-end.

Certain variants of algorithms or specialized implementations might be required when certain properties are back-end specific. In fact, in this work our node-level implementation of the Projected Gauss–Seidel (PGS) algorithm does not support neither thread-based parallelism nor GPU computations. However, we accelerate PGS by exploiting SIMD/AVX2 intrinsics. Our implementation is made for vector problems of size 4, as required by 3D phase-field problems (i.e., 1 component for the phase-field, 3 for the displacement). In each PGS sweep we perform operations on 4 components of the solution simultaneously (e.g., summations, matrix-vector, products, and checks on the inequality constraints).

**Fig. 1:**
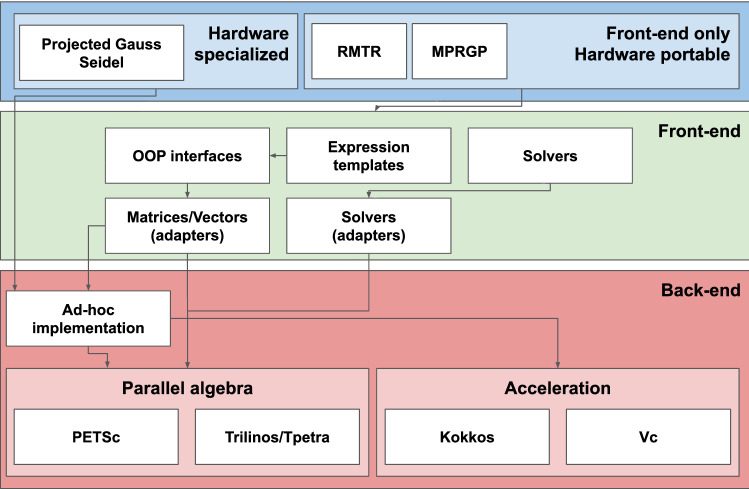
Layered architectural overview of the Utopia based algebra with focus on the RMTR method. The top layer represents the components for solving the fracture propagation problem. The middle layer represents the main design components which interface with the different back-end implementation at the bottom

The design overview of the algebra of our simulator is depicted in Fig. 1. Here, the top layer shows the components developed and tuned for this work. Such components are divided between front-end based, hence hardware portable, and specialized PGS component, targeting CPU hardware. The PGS component would have to be adapted or substituted for using the library with GPU nodes. The middle layer is the front-end which consists of interfaces, object oriented programming (OOP) abstractions, adapters, and front-end based algorithms. The bottom layer is where all the actual functionalities are implemented either by means of existing library, or ad-hoc extensions.

### Finite element assembly

Our current implementation of the finite element model phase-field fracture model targets CPU architectures. The code relies on the Utopia–PETSc tensors for the algebra in combination with the PETSc DMDA package for creating the hierarchy of structured grids and initializing matrices and vectors. The PETSc DMDA is encapsulated and used exclusively for steps requiring MPI communication, such as local-to-global or global-to-local operations. In this implementation the hierarchy of grids is generated by uniform refinement and the elements are uniform quadrilaterals in 2D and hexahedra in 3D. On each level of mutlilevel hierarchy, we discretize the minimization problem (6) using first order tensor-product finite elements.

In phase-field for fracture problems, a significant part of the computational time is dedicated to performing numerical quadrature and assembling Hessian matrices. In order to reduce the footprint of this routine we performed three steps to optimize its computation.

First, since we are exclusively using structured grids, we pre-compute several quantities for one element and reuse them for all elements. In fact, we pre-compute all quantities associated with the model and discretizations that are uniform between elements. These include all test-space related quantities such as shape-functions, gradients, strains, principal strains and stresses, and geometric quantities such as Jacobian matrices and determinants.

Second, we ensure that most loops can be unrolled by providing the compiler with compile time loop-ranges, such as spatial dimension, and number of shape-functions of an element.

Third, we developed quadrature routines based on *single instruction multiple data* (SIMD) intrinsics. We integrated the Vc library (Kretz and Lindenstruth 2012) for having a portable abstraction for different vector instruction sets (e.g, AVX, AVX2, and SSE2). Note that in our implementation we use double precision floating point numbers on CPUs supporting AVX2 instruction sets, which allows us to exploit 4 SIMD lanes.

We developed small tensor types which are designed to be used within assembly kernels and naturally integrate with Vc abstractions. With the goal of keeping the code usable and readable

we use operator overloading for representing algebraic operations. As a consequence, in order to avoid copies we implemented in-line operations using expression templates. Here, each in-line operation consumes or produces a Vc vector object.

3D scenarios require a 27-points quadrature rule. This allow us to perform quadrature using 7 vectorized quadrature points/weights by wasting only one SIMD lane. The vectorized version of the quadrature routines displays a speed-up of approximately $2.7\times$ with respect to the standard version.

**Fig. 2:**
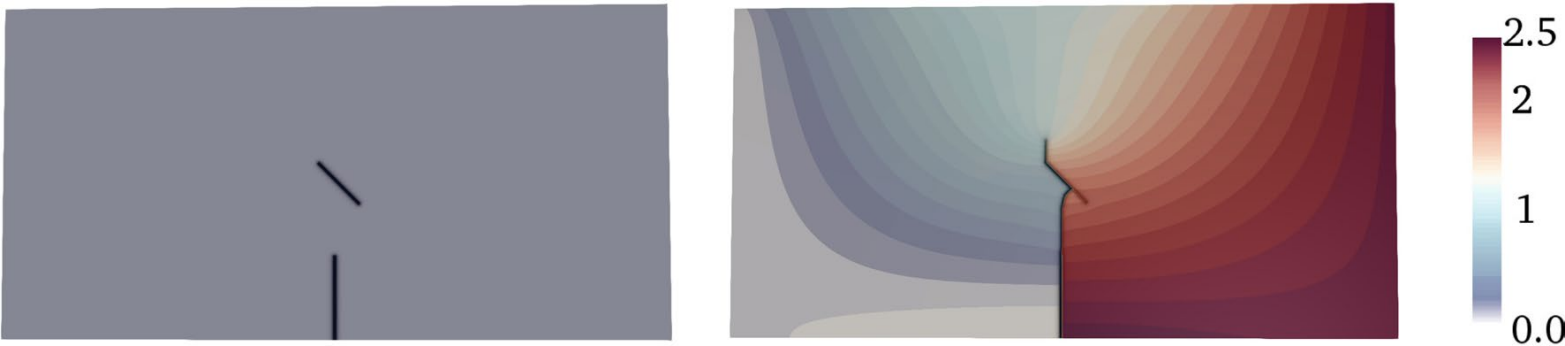
Tension test of asphalt specimen: two-dimensional simulation with 2 fractures and 949, 227 degrees of freedom. Color represents displacement field in mm

## Numerical experiments

We demonstrate the efficiency of the proposed phase-field fracture simulation framework using four numerical examples. First two example are used to validate our code using experimental measurements and analytical computations. Then, we consider more complicated scenarios of pressure induced fracture propagation of stochastic fracture networks, inspired by hydraulic simulations performed in enhanced geothermal systems.

We prescribe initial fractures by setting *c* to its transitional state from intact to broken, we check if the nodal position ${\mathbf {x}}$ lies inside of a parametric fracture description, then we mark the related parts of the domain as broken. This is done by setting the nodal coefficient for the phase-field variable to be equal to 1. Otherwise, we mark the material as intact by prescribing the nodal value of the phase-field to be equal to 0. The value of the length-scale parameter $l_s$ is set up as $l_s=2h$, where *h* denotes the mesh size, for all presented numerical examples.

Unless specified differently, we terminate the RMTR method, when the following stopping criterium is satisfied:

14 $$\begin{aligned} \mathcal {E}({\mathbf {x}}_i, f)< 10^{-6} \quad \text {or} \quad \Vert {\mathbf {x}}_i - {\mathbf {x}}_{i-1} \Vert < 10^{-12}, \end{aligned}$$

where ${\mathbf {x}}_i$ denotes the current iterate, defined on the finest level. The criticality measure $\mathcal {E}({\mathbf {x}}, f)$ is defined as $\mathcal {E}({\mathbf {x}}, f):= \Vert \mathcal {P} ({\mathbf {x}}- \nabla f({\mathbf {x}})) - {\mathbf {x}}) \Vert$, where $\mathcal {P}$ is an orthogonal projection onto the feasible set (Gratton et al. 2008a).

The main output data of the experiments can be downloaded from the Zenodo online repository (Zulian et al. 2020).

### Validation

In this section we describe the numerical simulations performed to validate the proposed software library. First, we consider a crack propagation in an asphalt specimen and compare the numerical solution with experimental data. Second, we present a three-dimensional benchmark to validate our code against an analytical solution. Finally, we introduce both two-dimensional and three-dimensional scenarios with stochastic fracture distributions to demonstrate the capability of our code to deal with large-scale simulations.

#### Tension test of asphalt specimen

We consider two initial cracks inserted in an asphalt specimen. The initial crack length is set equal to $a = 5\,\text {mm}$, the initial width is set equal to $w_0=0.2\,\text {mm}$, whereas the relative positions of the two cracks is defined such that they comprise an angle equal to $45\,^\circ$. The background matrix is a two-dimensional rectangle with height equal to $20\,\text {mm}$ and width equal to $40\,\text {mm}$. The Lamé parameters of the asphalt are $\mu = 2.23\,\text {N/mm}^2$ and $\lambda = 3.35\,\text {N/mm}^2$, whereas the fracture energy is set equal to $\mathcal {G}_c=0.270\,\text {N/mm}$ in agreement with (Hou 2014). Concerning the boundary conditions, we fix the left side of the rectangle whereas an incremental displacement is applied on the right side and defined as $u(t)=u_0+\varDelta t u_0$ with $u_0=3.0\,\text {mm}$ and $\varDelta t =0.01\,\text {s}$.

In Fig. 2 we show the initial and final configuration, where the two fractures interact with each other. Here, the mean displacement reached on the right side of the sample, $u=2.366\,\text {mm}$, corresponds to a critical load $\sigma _{c}^{n}=0.343\,\text {MPa}$, in good agreement with the experimental result $\sigma _{c}^{e}=0.30\,\text {MPa}$ reported in Hou et al. (2015).

#### Sneddon test of pressure induced fracture

**Fig. 3:**
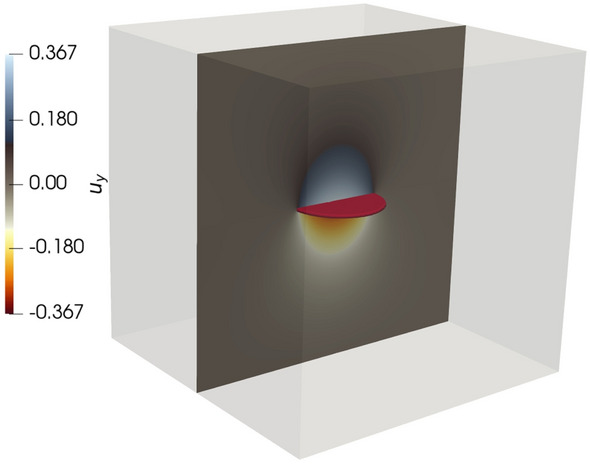
Sneddon test: crack opening and displacement (*y*-direction). The red color illustrates the fracture iso-surface for $c=0.9$

Following Sneddon and Lowengrub (2013), Yoshioka and Bourdin (2016), we validate our simulation framework by considering a horizontal penny-shape fracture embedded into domain $\varOmega :=(-10, 10)^3$. The penny is centered around the origin and has the radius $r:=3$. The crack opening displacement (COD) and total crack volume (TCV) of the internally pressurized fracture can be analytically computed as

$$\begin{aligned} \text {COD}({\mathbf {x}})&:= \frac{4 p r (1-\nu ^2)}{\pi E} \sqrt{1 - \bigg (\frac{\Vert {\mathbf {x}}\Vert _{\ell _2}}{r}\bigg )^2} \\ \text {TCV}&:= \frac{16 \pi p r^3 (1-\nu ^2)}{3 E}, \end{aligned}$$

where *E* is a Young’s modulus, $\nu$ denotes Poisson’s ratio, *p* stands for pressure and ${\mathbf {x}}$ denotes coordinates of a given point. In performed experiment, we set $E=1$, $\nu =0.2$, $p=0.1$ and $\mathcal {G}_c=1$, i.e., TCV equals to 13.824 and value of COD at the origin is 0.366. Figure 3 depicts the simulation result.

Table 1 depicts the error of the TCV and COD with respect to the increasing degree of freedom and decreasing value of the length-scale parameter. Recall, that in the numerical simulations, the condition $l_s>h$ has to be satisfied. As we can see, the phase-field approximation converges as $h\rightarrow 0$ and $l_s \rightarrow 0$. We would also like to highlight, that to obtain an accurate solution, simulation with a large number of dofs/computational power is required.

**Table 1:** Error of the TVC and COD for Sneddon test as a function of degrees of freedom and length-scale parameter $l_s$

$\#$ Dofs.	$l_s$	Err-TCV	Err-COD
202,612	1.08	$70.08\%$	$20.85\%$
1,826,132	0.52	$17.80\%$	$7.91\%$
15,479,572	0.25	$5.48\%$	$3.19\%$
127,420,052	0.13	$0.34\%$	$0.48\%$
434,125,332	0.08	$0.055\%$	$0.29\%$

### Pressure induced fracture propagation of stochastic fracture networks

In this section, we study the pressure-induced fracture propagation of stochastic fracture networks in two-dimensional and three-dimensional scenarios. The problems of this type occur in several geoscience applications, e.g. hydraulic fracturing Wick et al. (2016). Here, we demonstrate the applicability of the phase-field approach for such scenarios by considering large-scale problems with 1000/100 fractures in two/three-dimensions, respectively. To our knowledge, this is the first time that any phase-field fracture simulation framework was employed to handle such complex scenarios.

We generate the pre-existing fracture networks using a two-stage process. First, we describe each fracture as a one-dimensional object with a randomly assigned hypo-center, orientation, and length. In particular, we employ a uniform distribution to place the hypo-centers over the entire domain and assign their orientation to a value between $-80 ^\circ$ and $80 ^\circ$. The fracture length is drawn from a scale-invariant power-law distribution (de Dreuzy et al. 2001), defined as $n(l) = l^{-a}, \ \ \text {for} \ l \in [l_{\text {min}, \text {max}}],$ where *n*(*l*)*dl* represents a number of fractures with size belonging to the interval $[l, l+dl]$, and $a \in [1,3]$ is the power-law length exponent. The symbols $l_{\text {min}}$ and $l_{\text {max}}$ denote the minimum and maximum fracture length, respectively. Performed experiments employ $\alpha =2.7$, $l_{\text {min}} = 0.2\,\text {mm}$, and $l_{\text {max}}=5\,\text {mm}$ in two-dimensions. In three spatial dimensions, we employ $\alpha =2.7$, $l_{\text {min}} = 0.2\,\text {mm}$, and $l_{\text {max}}=0.7\,\text {mm}$. In addition, we consider orientation along the third dimension, drawn uniformly from $-80 ^\circ$ to $80 ^\circ$, and fracture depth, which we set to 0.1.

In the second stage, each fracture is regularized through a volumetric representation with artificial width *w* proportional to the mesh size *h*, where $w=2\,h$. Hence, the resulting fracture networks consist of *smooth rectangle/parallelepipeds* randomly embedded in the surrounding matrix. The fracture network represents the initial datum for the phase-field parameter which evolves during the simulation depending on the prescribed pressure and the boundary conditions. To ensure, that the proposed benchmarks are replicable, the coordinates defining the initial fracture networks can be downloaded from our Zenodo repository (Zulian et al. 2021).

**Fig. 4:**
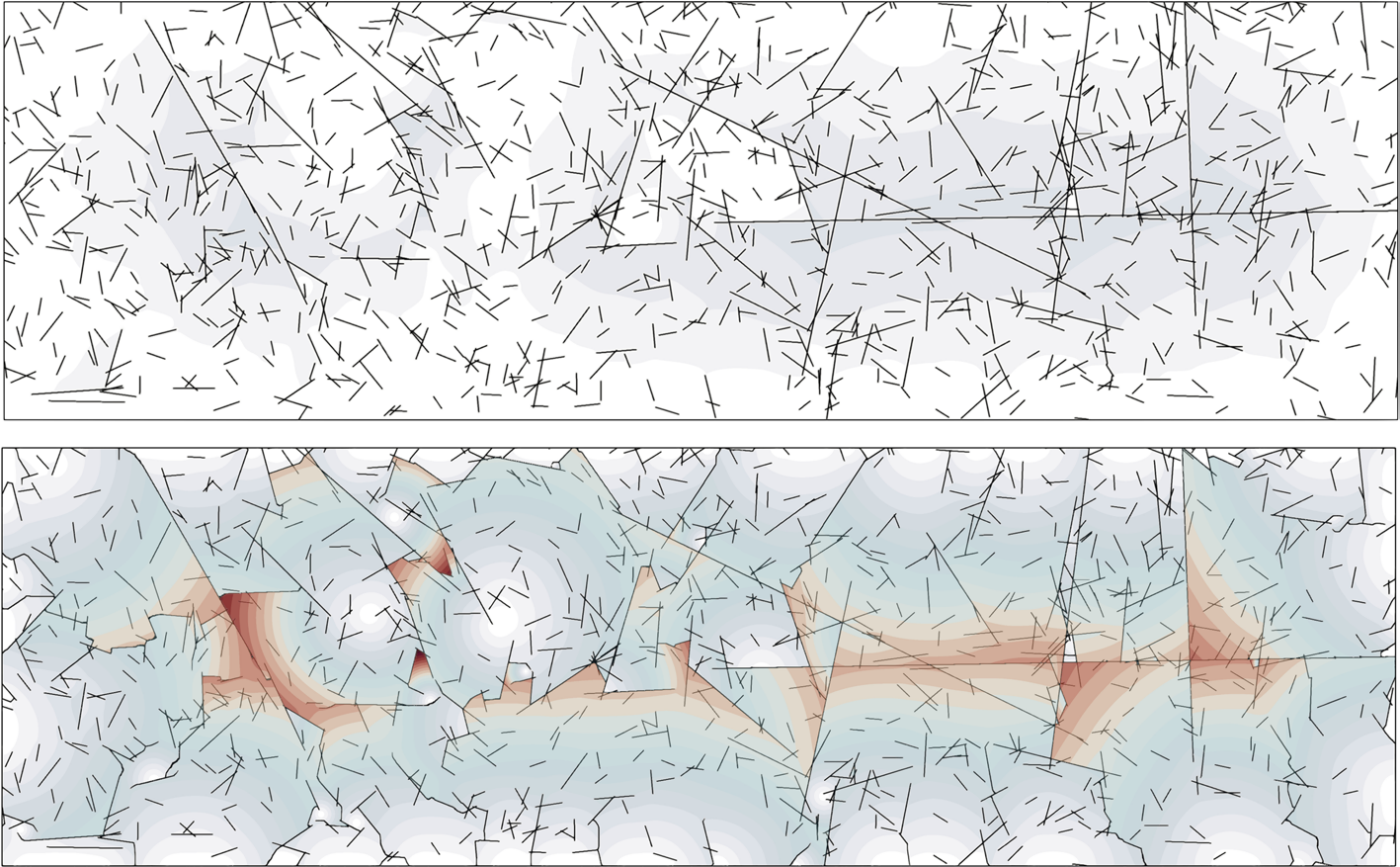
Two-dimensional simulation with 1000 fractures and 13,565,475 degrees of freedom. The colored overlay represents the displacement magnitude [0, 1.5] mm from transparent blue to opaque red. Top: initial fracture network. Bottom: final configuration

Our two-dimensional experiment considers a rectangular domain of size $3\,\text {mm} \times 10\,\text {mm}$. We construct a fracture network by generating 1000 initial fractures. During the whole simulation, we apply zero Dirichlet boundary conditions for the displacement field on all four sides of the domain. A pressure load is linearly increased at each loading step and defined as ${p(t) =p_0 + \varDelta t p_c}$, with ${p_0= 10^{-3}\,\text {GPa}}$, $\varDelta t=1\,$s and $p_c={p_0= 10^{-3}\,\text {GPa}}$. The initial setup and the simulation result are depicted in Fig. 4.

**Fig. 5:**
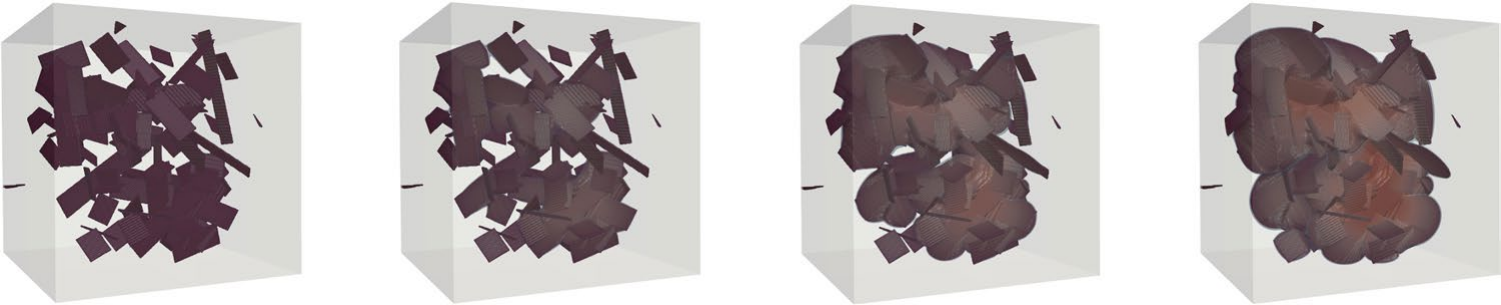
Large 3D fracture network: four loading steps of a three-dimensional simulation with 100 randomly distributed fractures and 242,793,828 degrees of freedom (number of levels is 4). The fracture iso-surface is displayed for $c=0.9$. The colored transparent overlay represents the displacement magnitude [0, 0.0032] mm from blue to red. Snapshots taken at different times $t \in \{0, 0.75, 0.8, 0.9 \}$ s

Our three-dimensional experiment considers a fracture network embedded in cube of size $1\,\text {mm} \times 1\,\text {mm} \times 1\,\text {mm}$. The initial set-up of the simulation takes into account 100 randomly distributed fractures as shown in Fig. 5. We apply zero Dirichlet boundary conditions for the displacement field on all sides of the domain. A pressure load is linearly increased at each loading step and defined as $p(t) =p_0 + \varDelta t p_c$, with $p_0=10^{-5}\,\text {GPa}$, $\varDelta t= 0.05\,\text {s}$ and $p_c=10^{-3}\,$GPa. Figure 5 depicts the evolution of the fracture network. For both experiments, we set the critical energy release to $\mathcal {G}_c = 1 \text {N/mm}$, whereas the Lamè parameters are set equal to $\lambda = 100{,}000 \,\text {N/mm}^2$ and $\mu = 100{,}000\,\, \text {N/mm}^2$, respectively, and describe the mechanical response of granite material Yu et al. (2018).

## Performance and scaling

All experiments have been performed at the Swiss National Supercomputing Centre (CSCS) with the Piz Daint super-computer on XC50^1^ or XC40^2^ compute nodes.

Every experiment uses all 12 cores of a XC50 node and, alternatively, all 36 cores of a X40 node, without hyperthreading. Thus, experiments running on 4 nodes are in fact running with $12\times 4=48$ and $36 \times 4 = 144$ MPI processes, respectively.

We traced the code to understand its parallel behaviour using mpiP (Vetter and Chambreau 2014) on XC40 compute nodes. Subsequently, we have run a test with a grid of $40\times 40\times 40$, 4 levels totaling 122.6 Million dofs over 1152 MPI tasks on the finest grid. Among all MPI calls, $79\%$ of the MPI time is due to three calls: AllReduce, Iprobe and Test. AllReduce calls are the most demanding. The heaviest are called in the calculation of the norms in the QP solvers, they count for $50\%$ of the MPI time and the $16\%$ of the overall application’s time. Following the reductions, Iprobe and Test calls, which are called by the matrix assembly, are noticeable for roughly $7\%$ of the MPI time.

### Algorithmic scalability

In this section, we investigate the algorithmic scalability of the proposed fracture simulation framework based on the RMTR method. We focus on the convergence properties of the RMTR method with respect to the number of degrees of freedom and number of processes. The comparison with the single level trust-region method as well as with the standard alternate minimization can be found in Kopaničáková and Krause (2020). As it has been demonstrated, the RMTR method can achieve a speedup of factor 2–8, in terms of computational time, compared to widely used alternate minimization on standard benchmarks. In addition, the sensitivity of the method with respect to the choice of the model parameters, such as degradation function can be found in Bilgen et al. (2019).

**Table 2:** Average number of V-cycles over all time-steps as a function of number of dofs and length-scale parameter $l_s$

$\#$ dofs	149, 307	605, 787	2, 440, 347	9, 795, 867
$l_s$	0.25	0.12	0.06	0.03
$\#$ V-cycles	79.2	106.4	131.3	149.5

The experiment performed using Asphalt tension test, RMTR setup with three levels

We investigate the convergence properties of the RMTR method for an increasing number of processors, for problems of a fixed size. The algorithmic scalability of the RMTR method is restricted by the choice of the trust-region subproblem solver (constrained QP-solver) employed on each level of the multilevel hierarchy. In this work, we employed the HJPGS method, see also Sect. 3.2.1. The convergence properties of the HJPGS method deteriorate with an increasing number of processors. This causes an increase in the number of V-cycles required by the RMTR method to converge to the desired tolerance, see Table 3. We have investigated the performance of the method using alternative QP-solvers, such as the projected conjugate gradient method (MPRGP, Dostál 2016). In this particular case, the number of required V-cycles remains stable with an increased number of processors. However, the soothing properties of the MPRGP method are significantly worse than those of the HJPGS. As a consequence, the RMTR method configured with the MPRGP solver performed worse, compared to the RMTR method configured with the HJPGS smoother.

Furthermore, we study the convergence properties of the RMTR method with respect to the increasing number of dofs. The conducted experiment considers the Asphalt tension test and the RMTR method configured with three levels. To mitigate the effects of the scalability of the HJPGS method on the obtained results, the experiment was run in serial. As we can see from Table 2, the average number of V-cycles increases together with the number of dofs. This is not surprising, as the value of the length-scale parameter $l_s$, tied to the refinement level, determines how accurately we can approximate sharp fracture surface. Thus, as we refine, we are capable of approximating the sharp fracture surface more accurately. However, the non-linearity and ill-conditioning of the underlying problem become more prevalent, which causes an increase in the required V-cycles.

**Table 3:** Effects of HJPGS convergence on performance

$\#$ nodes	4	8	16	32	$\#$ nodes	25	50	75	100
$\#$ V-cycles	126	135	147	154	$\#$ V-cycles	42	44	54	69

Average number of V-cycles over all time-steps as a function of number of nodes. Left: two-dimensional experiments performed with 1000 fractures and 28.7 mil. dofs. Right: three-dimensional experiment performed with 100 fractures and 122.7 mil. dofs. The experiments were performed using XC40 nodes

### Scaling measures

We analyze the performance of our code with strong and weak scaling measures. In *strong scaling* experiments the size of the problem fixed and the speed-up is measured when increasing the number of compute nodes. In particular, the parallel efficiency is defined as $e=\frac{T_b n_b}{T_n n}$ with $T_b$ being the base experiment’s runtime and $T_n$ being the experiment’s runtime on *n* nodes. The minimal number of nodes $n_b$ is chosen in such a way that the experiment fits into the node’s RAM.

In *weak scaling* experiments the global size of the problem is changed proportionally to the number of compute nodes. This is done such that the size of the sub-problem assigned to one node is kept fixed. Here, the parallel efficiency is defined as $e=T_b/T_n$ with $T_b$ being the base experiment’s runtime and $T_n$ being the runtime of the experiment on *n* nodes

For most experiments we analyze one of the reasons for the loss in scaling by employing the following measure of *imbalance*:

$$\begin{aligned} I(\text {method}) = \frac{ \left( \displaystyle \max _r T(r, \text {method}) - \displaystyle \min _r T(r, \text {method})\right) }{ \displaystyle \max _r T(r, \text {Total})}, \end{aligned}$$

where *T* is the computing time with respect to MPI rank *r* and any “method” of interest. The imbalance is measured independently for each run. Methods of interest are typically the ones with high variance in computing time. In all studies we will look at the imbalance of the most intensive routines the hybrid Jacobi projected Gauss–Seidel (HJPGS) solver, the Hessian local matrix assembly (L), and the Hessian local to global routine (G) where the data is communicated for the elements at process boundaries.

### 3D Sneddon test: a scaling study

In this section, we use the Sneddon test introduced in Sect. 5.1 for studying the scaling properties of the code. We keep the length-scale parameter $l_s=1.08$ fixed for all runs. This test case has the advantage of providing an analytical solution, and it is simple enough to be reproduced in future studies with different software stacks. Additionally, the problem is solved with 6 nonlinear iteration for any parallel configuration, hence keeping the required computing budget low even for large experiments. These experiments are run on the XC40 nodes of Piz Daint with 36 MPI processes per node. At every nonlinear iteration, we perform 2 pre- and post-smoothing steps.

*Strong scaling*: We conducted three strong scaling experiments with different grid resolutions.

**Fig. 6:**
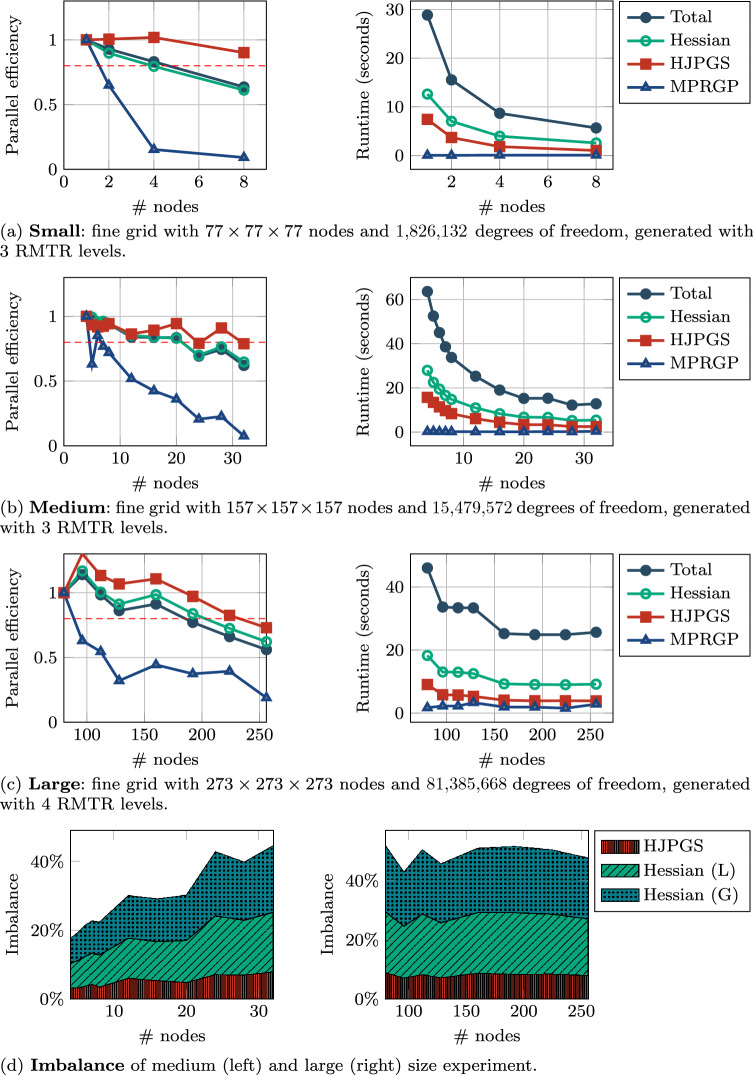
Sneddon test: small (**a**), medium (**b**), and large (**c**) experiments for measuring *strong scaling* efficiency and runtimes for the different components of our RMTR implementation. The horizontal dashed red line marks 80% efficiency. Imbalance (**d**) affecting the scaling efficiency of the implementation

Figure 6a illustrates how the software performs for a small experiment with a coarse grid of size $20 \times 20 \times 20$. Here we use 3 RMTR levels hence having a fine grid of size $77 \times 77 \times 77$ and a nonlinear problem with 1,826,132 dofs. The experiment was performed with 1, 2, 4, 8 nodes, and the run time is reduced from over a minute to a few seconds.

Figure 6b illustrates how the software performs for an experiment with an order of magnitude more degrees of freedom. Here, we have a coarse grid of size $40 \times 40 \times 40$, 3 RMTR levels, a fine grid of size $157 \times 157 \times 157$, and 15,479,572 dofs. The runs have been performed on 4, 5, 6, 7, 8, 12, 16, 2, 24, 28, 32 nodes. Figure 6d illustrates the imbalance which reduces the overall parallel efficiency. The hierarchy of grids is generated by refinement, hence any initial slight imbalance in coarse grid distribution is amplified each refinement. This difference in workload is particularly evident in the routine computing Hessian matrix. This routine is split into two phases: (1) the “local” assembly (L) where numerical quadrature is performed and the entries are added in the respective rows of the matrix; (2) the local to global routine (G), where entries on the boundary of the subdomains that belong to other processes are redistributed. The second phase could be avoided by leveraging overlapping decomposition of the grid hence avoiding this communication step. However, this contribution is restricted to non-overlapping domain decomposition techniques.

Figure 6c illustrates a large experiment with a coarse grid of size $35 \times 35 \times 35$, 4 RMTR levels, a fine grid of $273 \times 273 \times 273$, and 81,385,668 dofs. Here, we used 80, 96, 112, 128, 160, 192, 224, 256 nodes. This experiment differs from the previous two, due to the extra level of refinement, which reduces the cost of the coarse grid solver but will accentuate the imbalance as it can be observed in the measurements illustrated in the right-side area plot in Fig. 6d.

**Fig. 7:**
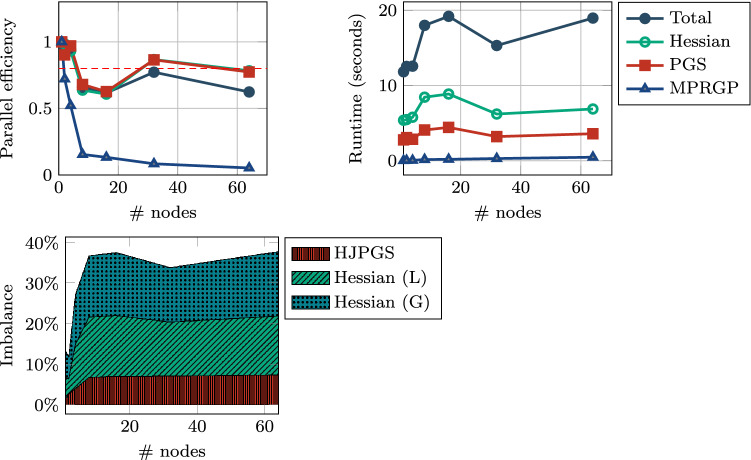
Sneddon test: *weak scaling* efficiency, runtimes, and *imbalance*. The size of the grid is $s \times s \times s$, where $s = \left\lceil (1000 \times n)^\frac{1}{3} \right\rceil$ hence with $s^3 \times 4$ dofs in the coarse level and $((s-1) \times 2^{(l-1)} + 1)^3$ at each RMTR level *l* (fine level is $l = 3$). The reminder rounded by the ceiling operator $\lceil \cdot \rceil$ will cause some fluctuations in the amount of work of each run and defects in the efficiency plot. The size of smallest run consists of 740,772 dofs while the largest one consists of 48,035,956 dofs

*Weak scaling:* Figure 7 illustrates the weak-scaling properties for runs with a relatively small amount of dofs per process (approximately 20,000). We ran the experiment on 1, 2, 4, 8, 16, 32, 64 nodes. The runtime is stable below 20 seconds and the main routines of interest in our implementation are stable below 10 seconds. It can be observed that the imbalance measures is a quite significant past the base run on one node. Except for MPRGP, which solves the coarse grid problem with negligible computational time, the scaling efficiency fluctuates around $80\%$

### Large 3D fracture network: a scaling study

In this section, we study the nonlinear multilevel operator, when used for solving the three-dimensional pressure-induced fracture propagation of the stochastic fracture network, described in Sect. 5.2. Scaling experiments were obtained by performing a single nonlinear solve which required a fixed number of V-cycles (10). However, the obtained results are conclusive, as the operations performed within a V-cycle are called repetitively during the whole simulation. The experiments were performed on the XC50 nodes of Piz Daint with 12 MPI processes per node.

*Strong scaling*: We conducted two strong scaling experiments one *small* with a coarse grid of $25 \times 25 \times 25$, 4 levels totalling 28.7 million dofs on the finest grid and one *large* experiment with a coarse grid of $40 \times 40 \times 40$, 4 levels totalling 122.7 million dofs on the finest grid. The small experiment was run on 4, 5, 6, 7, 8, 12, 16, 20, 24, 28, 32 nodes, the big one on 40, 48, 56, 64, 80, 96, 112, 128, 160, 192, 224, 256. Moreover, in both the two scenarios we employed the Hessian lagging strategy to reduce the numbers of times when Hessian matrix is assembled.

**Fig. 8:**
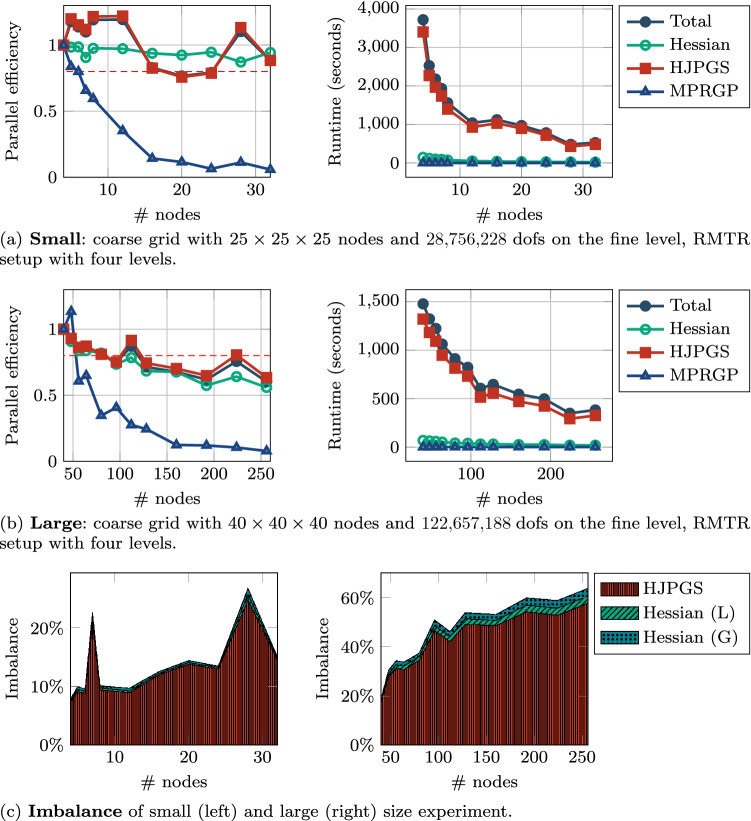
Large 3D fracture network: small (**a**), and large (**b**) experiments for measuring strong scaling efficiency and runtimes for the different components of our RMTR implementation. The horizontal dashed red line marks 80% efficiency. Imbalance (**c**) affecting the scaling efficiency of the implementation

In Fig. 8a we analyze the small experiment starting with $n_b=4$ nodes. Here, we depict the total parallel efficiency and the total run-time together with the parallel efficiency and the run-time of the routines which most affect the overall performance of the software library.

In Fig. 8b the same analysis is presented for the large experiment starting with $n_b=40$ nodes. Both the figures show that the total parallel efficiency oscillates depending on the number of nodes. This is due to slight imbalances illustrated in Fig. 8c which appear to have sometimes a bigger effect on the total runtime than with the same coarse grid size but a different node count. However, a comparison between the three-dimensional pneumatic scenarios and the 3D Sneddon test reveals that the use of the Hessian lagging strategy allows reducing the time invested in evaluating the Hessian and reserve it for solving the QP problem. In Table 4 we report the number of calls of the most relevant routines. We can observe that when parallelism is increased the HJPGS convergence effects discussed in Sect. 6.1 are sensibly changing the algorithmic behaviour of RMTR and affecting the scaling efficiency.

**Fig. 9:**
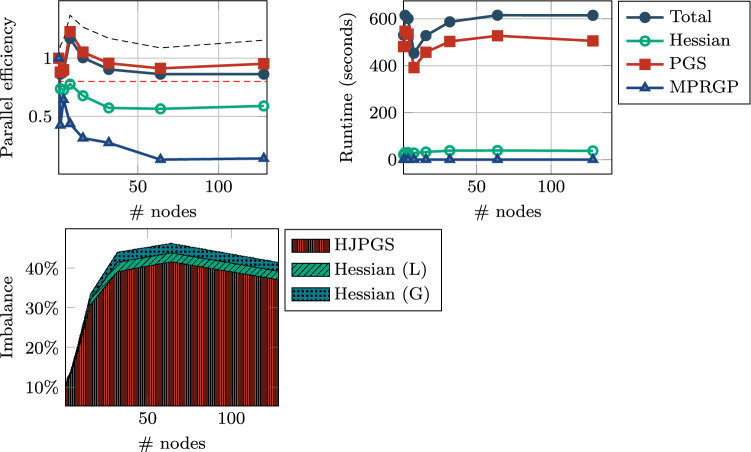
Large 3D fracture network: *weak scaling* efficiency, runtimes, and *imbalance*. The size of the grid is $s \times s \times s$, where $s = \left\lceil (729 \times n)^\frac{1}{3} \right\rceil$ hence with $s^3 \times 4$ dofs in the coarse level and $((s-1) \times 2^{(l-1)} + 1)^3$ at each RMTR level *l* (fine level is $l = 3$). The reminder rounded by the ceiling operator $\lceil \cdot \rceil$ will cause some fluctuations in the amount of work of each run and defects in the efficiency plot. The size of smallest run consists of 1,098,500 dofs while the largest has 188,183,524 dofs. The dashed black line represents the “corrected” efficiency

*Weak scaling*: For weak scaling, we have set up the experiment with a coarse grid of $9 \times 9 \times 9$ on a single node and incremented then by doubling the nodes and adapting the dimensions to have a similar number of dofs on the coarse grid. Experiments with a cube number of nodes are exact in the sense that the work per node on the coarse grid is the same as for the base experiment on one node. In Fig. 9c we can see the results for the parallel efficiency where we have a sub-grid size of $10 \times 10 \times 10$ on each node. Additionally,we have a dashed black line which gives us an upper estimate of the parallel efficiency. It is a “corrected” value where we multiply *e* with a constant $c=\frac{N}{N_bn}$ with *N* being the number of dofs on the finest grid and $N_b$ being the number of dofs on the finest grid for the experiment on one node. This correction factor is larger than 1, because doubling each dimension on the coarse grid will increase the number of dofs by a factor larger than 8 on the finest grid. For a setup with 4 levels, the number of dofs on the finest grid in *x*-direction is $8N_x-7$, similarly in *y* and *z*-direction, which results in a larger multiplication factor on the finest level than the multiplication factor on the coarse level.

**Table 4:** Large 3D fracture network: *large* experiment from Fig. 8

Nodes	Hessian	Gradient	Energy	HJPGS	MPRGP
40	39	111	127	65	8
48	36	96	113	59	7
56	37	99	118	62	7
64	37	100	117	61	7
80	42	103	167	95	10
96	42	113	148	80	8
112	40	107	137	73	8
128	39	99	138	76	8
160	43	104	173	99	10
192	43	106	170	96	10
224	38	105	123	63	8
256	44	111	178	100	11

Number of calls of routines and algorithms for different node configurations. The convergence rate of HJPGS deteriorates with more parallelism and it is affected by the domain decomposition. It can be observed that this aspects sensibly affects the number of calls of other methods

## Conclusion

We presented the first open-source code for numerical modelling of large-scale phase-field fracture simulations using the RMTR method. Our implementation of the phase-field fracture model employs an expression template-based assembler designed for structured grids and 2D/3D tensor-product finite elements. Our implementation of the RMTR method with its different components, such as the quadratic programming solvers, provided in the Utopia software library can deal with non-convex and geometrically complex problems in an efficient and scalable way. Every aspect of the code has been first optimized for single-core CPU performance, then improved for MPI-based parallelism.

All the numerical examples show the capabilities of our simulation framework and its suitability for large-scale geoscience applications, such as hydraulic fracturing of complex fracture networks. To this end, our studies show the parallel performance by analyzing strong and weak scaling properties to the limits of the standard PETSc configuration, i.e., with 32-bit indices.

We performed two scaling studies with different RMTR set-ups where the first, based on the Sneddon test, has a simple set-up and the second, the large fracture network experiment, is more complex due to a large number of fractures. We differentiated the large fracture network experiment by using the Hessian lag strategy. Here, we observed how the weight of the computation is moved from quadrature to linear algebra.

The current implementation of both discretization and model is tailored towards CPU based computing architectures. However, we point out that most of this code has been prepared already with the perspective to be ported to GPU based computing architectures. To achieve this goal there are however two main challenges. First, the implementation of the quadrature rules which, due to the limited memory available and the GPU work model, requires specific design measures. Second, the HJPGS algorithm has to be either ported to GPU [using independent-set coloring (Zhang 1996)], or a more suitable alternative with equivalent smoothing properties has to be found. We emphasize that for remaining parts of our multilevel solver we can instead just switch to the back-end which targets GPUs, the Utopia/Tpetra backend. Results presented in this work are foreseen to be used for comparisons with future GPU accelerated versions of this code.

In this work we focused on networks with high fracture density, which represent a challenging class of problems due to the complex geometry and the non-convexity of the underlying minimization problem. Future work shall include to port the entire framework to GPU architectures, and the integration of adaptive octree data structures [e.g., by using DMPlex or P4est (Burstedde et al. 2011)] to efficiently handle the discrete representations of sparse fracture networks.

## Footnotes

1 A XC50 node consists of one Intel ^®^ Xeon ^®^ E5-2690 v3 @2.60 GHz (12 cores, 64 GB RAM)

2 A XC40 node consists of two Intel ^®^ Xeon ^®^ E5-2695 v4 @2.10 GHz ($2 \times 18$ cores, 64/128 GB RAM)
